# Reliability of a Novel Hematological Malignancy Specific Patient-Reported Outcome Measure: HM-PRO

**DOI:** 10.3389/fphar.2020.571066

**Published:** 2020-10-20

**Authors:** Pushpendra Goswami, Esther N. Oliva, Tatyana Ionova, Roger Else, Jonathan Kell, Adele K. Fielding, Daniel M. Jennings, Marina Karakantza, Saad Al-Ismail, Graham P. Collins, Stewart McConnell, Catherine Langton, Magda J. Al-Obaidi, Metod Oblak, Sam Salek

**Affiliations:** ^1^School of Life and Medical Sciences, University of Hertfordshire, Hatfield, United Kingdom; ^2^Hematology Unit, Grande Ospedale Metropolitano, Reggio Calabria, Italy; ^3^St. Petersburg State University Medical Center and Multinational Centre for Quality of Life Research, St. Petersburg, Russia; ^4^Patient Research Partner, Milton Keynes, United Kingdom; ^5^Department of Hematology, Cardiff and Vale University Health Board, Cardiff, United Kingdom; ^6^Department of Hematology, University College London Cancer Institute, London, United Kingdom; ^7^Department of Hematology, Royal Surrey County Hospital NHS Foundation Trust, Guildford, United Kingdom; ^8^Department of Hematology, Leeds Teaching Hospitals NHS Trust, Leeds, United Kingdom; ^9^Department of Hematology, Singleton Hospital, ABM University Health Board, Swansea, United Kingdom; ^10^Department of Hematology, Oxford University Hospitals NHS Trust, Oxford, United Kingdom; ^11^Department of Hematology, West Middlesex University Hospital, Isleworth, United Kingdom

**Keywords:** hematological malignancy, Hematology-Specific Patient-Reported Outcome Measure, quality of life, symptoms, reliability, internal-consistency, clinical practice, clinical research

## Abstract

**Background:**

Patients’ experience of symptoms often goes undetected during consultation in an outpatient clinic, and the use of a patient-reported outcome measure (PRO) in such a setting could be useful to aid treatment decision-making. A new PRO measure, the HM-PRO (Hematological Malignancy Specific Patient-Reported Outcome Measure) has been recently developed to evaluate hematological malignancy (HM) patients’ health-related quality of life (HRQoL) and their symptom experience in daily clinical practice as well as in research. The objectives of the study were to assess: the internal consistency of the scores for Part A (impact) and its four domains (physical behavior; social well-being; emotional behavior; and eating and drinking habits) and Part B (signs and symptoms); and the test-retest reliability of the individual items of the newly developed hematological malignancy specific composite measure, the HM-PRO.

**Methods:**

This was a prospective longitudinal observational study where 150 patients with different HMs and different stage of disease (male n = 98 (65.3%); mean age 64.9 ± 14.4 years, range 17.9–89.2 years; mean time since diagnosis 3.7 ± 4.9 years, range 0.04–25.8 years) completed the HM-PRO at baseline (assessment 1 at t1) and after 7 days (assessment 2 at t2). Data analysis was performed using IBMSPSS 23 statistical software.

**Results:**

The Cronbach’s alpha estimates of the HM-PRO for both assessment points (t1 and t2) were above 0.9 for Part A, and above 0.8 for Part B, showing strong stability of the measurement. The level of agreement for the reproducibility between the two assessments, using intra-class correlation coefficients (ICC), was very strong with Part A: ICC = 0.93 (95% CI = 0.90–0.95), and Part B: ICC = 0.91 (0.88–0.93). The ICC for the four domains of Part A ranged from 0.85–0.91. The ICC was greater than 0.8 for overall score of Part A and Part B for all the 10 diagnoses, confirming strong reliability.

**Conclusion:**

This study clearly indicates that the HM-PRO possesses strong test-retest reliability for both Part A and Part B. The Cronbach’s alpha confirmed acceptable internal consistency. The extensive reliability testing described in this study supports the generic nature of the HM-PRO for use in hematological malignancies in both routine clinical practice, to aid treatment decisions, as well as in research.

## Introduction

Hematological Malignancies (HM) include neoplasms of myeloid and lymphoid cell lines (HMRN, 2004), with an expected UK incidence rate of 38,740 per annum (HMRN, 2014). WHO defines the primary objectives of cancer diagnosis and treatment as the cure, prolongation of life and improvement of the quality of life (QoL) (WHO, 2017). With the advances in the treatment modalities, patients treated for HMs are able to survive their disease and evidence suggests that health-related QoL (HRQoL) of patients with HM is significantly affected by the disease and its treatments (Persson et al., 2001; Holzner et al., 2004; Santos et al., 2006; Mols et al., 2007; Shanafelt et al., 2007; Strasser-Weippl and Ludwig, 2008; Johnsen et al., 2009; Goswami et al., 2019a). Hence, maintaining a good QoL is of paramount importance in treating and caring for patients with HM.

A recent systematic literature review identified the health-related QoL (HRQoL) issues important to patients with HMs and the HRQoL instruments currently used in hematology (Goswami et al., 2019a). This review assessed the conceptual coverage of the identified HRQoL instruments as well as their measurement properties. It was reported that these instruments do not cover important HRQoL and highlighted the need for the development of a new PRO with a robust methodology (Goswami et al., 2019a). Similar finiding were reported by another systematic reviwer which focused on patients with multiple myeloma (Osborne et al., 2012; Goswami et al., 2019a). A new PRO measure, HM-PRO (hematological malignancy specific patient-reported outcome measure), has been recently developed to evaluate the HRQoL of patients with HMs in daily clinical practice as well as in research (Goswami et al., 2017; Goswami et al., 2020b; Goswami et al., 2020c).

Reliability is a measure of reproducibility of an instrument; it is the degree to which the instrument yields the same score each time it is administered, while the underlying construct remains the same. It is a way to reflect the amount of error inherent in the measurement of the HRQoL (Mcdowell and Newell, 1996; Streiner and Norman, 2003). The use of a reliable instrument in daily clinical practice settings is crucial. An unreliable instrument will lead to an error in the measurement of HRQoL eventually resulting in a misleading interpretation of the scores and its ultimate influence on clinical decision-making. The importance of PRO instruments in evaluating the impact of the disease and its treatment and in understanding the health outcomes is fully established (Dobrozsi and Panepinto, 2015). Patients’ experience of symptoms often goes undetected during consultation in an outpatient clinic, and therefore the use of a PRO instrument in such setting could be very useful to aid treatment decision-making. An unreliable patient-reported outcome measure (PRO) instrument will have a negative impact on the measurement of such symptoms. Poor reliability of an instrument would also affect its convergent validity, i.e., it may affect the correlation of the instrument with other measures (John and Soto, 2007) in an attempt to establish its being fit for purpose. However, a PRO instrument with acceptable reliability would result in a more accurate assessment (Mccrae et al., 2011) which, in turn, influences the ability of such instrument to detect change over time, i.e., its responsiveness. Hence, reliability is very central to the measurement properties of an instrument. There are three possible ways to measure the reliability: internal consistency; inter-rater reliability; and test-retest reliability.

Internal consistency is the measure of the homogeneity of the instrument. It reflects an instrument’s ability to identify variability within a patient population and extent to which all the items in the scale measure the same underlying concept (Streiner and Norman, 2003; Mccrae et al., 2011). The inter-rater reliability measures the agreement between the data collected from different raters. It is used to measure whether different raters are harmonious in their observations and scoring. The test-retest reliability focuses on the reproducibility of the scores. It assesses an instrument’s ability to measure the same construct at two different time points while keeping all other variables constant. In an ideal situation, the scores of an instrument, completed at two different points, should be the same, proving the reproducibility reliability of the instrument. The US Food and Drug Administration (FDA) assesses the stability of scores by using evidence of intraclass correlation coefficient (ICC) and the time period of assessment for test-retest test (USFDA, 2009). The objectives of this study, therefore, were to: (1) assess the internal consistency of the scores for Part A (impact) and its four domains, and Part B (signs and symptoms) of the HM-PRO; (2) assess the test-retest reliability of the individual items of the HM-PRO, the scores of the domains of Part A, and overall scores of Part A and Part B.

## Methods

### Ethics

Multicenter ethics approval was obtained from the National Research Ethics Service (NRES) South West Bristol, UK (ref 14/SW/0033) followed by individual “research and development” approvals from all the participating centers. A signed informed consent was obtained from all the study participants.

### Study Design

The study was a prospective longitudinal observational study. All study participants were assessed on two occasions, at baseline (t1—assessment 1) and after 7 days (t2—assessment 2). The time interval of 7 days between assessments was chosen to minimize the “learning effect” (Salek, 1992). The choice of 7 days period was further confirmed after discussing it with the patient research partner and the clinicians (hematologist) involved in the study design. The selection of the optimal time between the two assessments is very crucial as it will help in avoiding the overestimation (if it is shorter) and underestimation (if it is longer) of reliability. Patients who were receiving treatment or had completed their course of treatment may have a drastic change in their symptoms and HRQoL in a short period of time; hence, a slight difference in score was expected between the two assessments.

### Patient Population

Patients with different hematological malignancies were recruited from seven secondary care hospitals in the UK. The inclusion criteria were: diagnosed with hematological malignancy as per the most recent WHO classification; at any stage of disease (defined as stable, progressing, and remission)—confirmed by clinical staff; at any stage of the treatment (due to start the treatment, on treatment, or finished treatment); able to read and write in English; ability to give written informed consent; and aged 17 or above. The exclusion criteria were: patients whose diagnosis was not confirmed; aged below 17; unable to read and write in English; patients with compromised mental capacity; and unavailable to complete the second assessment after 7 days.

### Measurement Instrument

The HM-PRO, a hematological malignancy specific patient-reported outcome measure, is a newly developed composite measure consisting of two scales: Part A (impact) and Part B (signs and symptoms) (Goswami et al., 2016; Goswami et al., 2018a; Goswami et al., 2018b; Goswami et al., 2019b; Goswami et al., 2020c). Part A measures the impact of hematological malignancy and its treatment on a patient’s HRQoL, and Part B captures the severity of different disease symptoms and treatment side effects. Part A has a total of 24 items in four domains: physical behavior (7); social well-being (3); emotional behavior (11); and eating and drinking habits (3) rated on a 3-point Likert scale (0 = not at all, 1 = A little, and 2 = A lot), and “not applicable” as a separate response option. Part B consists of 18 items in a single domain, with 3-point severity Likert scale (0 = not, 1 = Mild, and 2 = Severe). The third items of the “Eating and drinking habits” domain in Part A, i.e., “*My drinking habits have changed*”, and the ninth item of Part B “*I have skin problems (e.g. itching, bruises, rashes, etc.)*” are not included in the scoring system due to misfit in Rasch model but were collected for additional information (Goswami et al., 2017; Goswami et al., 2018b). The HM-PRO has demonstrated good construct validity of both convergent and divergent type comparing it with existing closely and distantly related PROs (Goswami et al., 2020a).

### Procedure

During the first assessment, patients were approached by the clinic/research nurse in an out-patient/day-care or in-patient setting, who explained in brief about the study and asked their willingness to participate. Those who agreed were asked to complete the HM-PRO after reading the patient information sheet and giving written informed consent. After completing the first assessment, all the patients were provided with a package containing the HM-PRO for the second assessment and a freepost self-addressed envelope to return the completed instrument after 7 days. Other relevant patient demographic information and preferred contact details were collected. On the 6^th^ day following the initial assessment, patients were reminded *via* text/calls/emails and asked to complete the instrument on the 7^th^ day and use the freepost envelope provided in the pack to send it back to the research team.

### Data Processing and Analysis

The data entry was performed in Microsoft Excel and 20% entries were randomly selected for cross-validation by a reviewer. Cleaning, coding, and analysis of the data were performed using SPSS Windows version 23. Cronbach’s alpha was calculated to measure the internal consistency of the scales and subscales of the HM-PRO, which is the average inter-item correlation (Cronbach, 1951; Frost et al., 2007; Tavakol and Dennick, 2011; Devellis, 2016). The alpha value reflects the extents to which the instrument measures the concept consistently. Cronbach’s alpha value greater than 0.7 was taken as reliable (Tavakol and Dennick, 2011). Spearman’s rank correlation was also calculated to assess the inter-item and item-total partial correlation. A moderate correlation (r_s_ = 0.2) is expected between items (Streiner and Norman, 2003). Intra-class correlation was calculated to assess the level of agreement between scores from the first assessment (test) and after 7 days (re-test). An ICC value of 1 means that 100% variability is because of difference between patients, and the ICC value of 0 means that all variability is due to within-patient variability and error (Studenic et al., 2016). A 2-way mixed-effects, absolute agreement, multiple raters measurement type of ICC was chosen as per McGraw and Wong convention (Mcgraw and Wong, 1996).

## Results

### Patient Demographics

**Figure 1:**
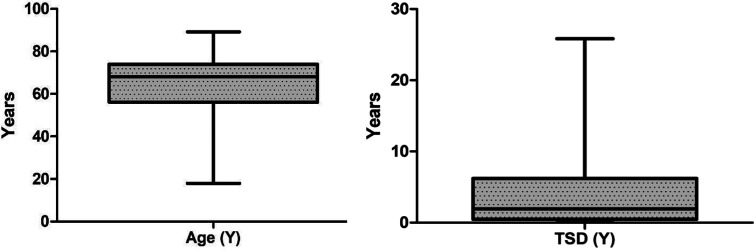
Box plot showing median values and interquartile range (IQR) for age and time since diagnosis (TSD).

A total of 193 patients with different hematological malignancies (acute lymphoid leukemia = 12; acute myeloid leukemia = 28; chronic lymphoid leukemia = 17; chronic myeloid leukemia = 13; multiple myeloma = 33; indolent non-Hodgkin lymphoma = 16; aggressive non-Hodgkin lymphoma = 22; Hodgkin lymphoma = 14; myelodysplastic syndrome = 15; and myeloproliferative neoplasm = 23), at different stage of disease (stable = 71; remission = 64; and progressing = 58) participated in the study and completed the first assessment (t1). The mean age of patients was 63.3 (± 15.2, range = 18–89 years) and the mean time since diagnosis was 3.8 (± 4.9) years. However, a total of 150 patients completed the second assessment after 7 days (t2) and returned them by post, with a response rate of 77.7%. The data from these 150 patients (mean age 64.6 ± 14.4 years, range 17.9 to 89.2; mean time since diagnosis 4.0 ± 5.1 years, range 15 days to 25.8 years) were used for the test-retest reliability analysis (**Table 1** and **Figure 1**). The highest number of patients (n = 41, 27.3%) were diagnosed less than 6 months prior to the study and the highest number of patients (n = 46, 30.7%) were in the age groups 70–80 years.

**Table 1:** Demographics characteristics of the study participants (n-150).

n = 150		Median	Range
**Age (Years)**		68.13	17.9–89.2
**Time since Diagnosis (years)**		1.9	0.04–25.84
		**n**	**%**
**Gender**	Male	98	65.3
	Female	52	34.7
**Ethnic Origin**	White	139	92.7
	Asian or Asian British	9	6.0
	Black British	2	1.3
**Inpatient/Outpatient**	Inpatient	9	6.0
	Outpatient	141	94.0
Disease Type*	ALL	9	6.0
	AML	19	12.7
	CLL	14	9.3
	CML	11	7.3
	MM	29	19.3
	INHL	11	7.3
	ANHL	17	11.3
	HL	11	7.3
	MDS	13	8.7
	MPN	16	10.7
**Stage of Disease**	Stable	58	38.7
	Remission	51	34
	Progressing	41	27.3
**Comorbidities**	Comorbidities, cases	45	30.0
	Other Cancer, cases	10	6.7
	No other condition	95	63.3

*ALL, acute lymphoid leukemia; AML, acute myeloid leukemia; CLL, chronic lymphoid leukemia; CML, chronic myeloid leukemia; MM, multiple myeloma; INHL, indolent non-Hodgkin lymphoma; ANHL, aggressive non-Hodgkin lymphoma; HL, Hodgkin lymphoma; MDS, myelodysplastic syndrome; MPN, myeloproliferative neoplasm.

### Internal Consistency

The Cronbach’s alpha estimates of the HM-PRO for assessments 1 and 2 (at t1 and t2) were above 0.9 for Part A, and above 0.8 for Part B, showing strong instrument stability. For individual domains of Part A, the lowest alpha value was observed for the social well-being domain for assessment 1 (alpha = 0.70) and assessment 2 (alpha = 0.68). Alpha value was greater than 0.8 (i.e., both assessments) for Physical Behavior, Emotional Behavior, and for Eating and drinking habits. Optimal homogeneity is reflected in moderate inter-item correlation and moderate-to-strong corrected item-total correlations (Streiner and Norman, 2003); hence, this was also examined. The corrected item-total correlation (CITC) of Part A items ranged from 0.42 to 0.75 for assessment 1 and 0.40 to 0.80 for assessment 2, reflecting strong internal consistency. For assessment 1, the lowest correlation was seen for Item “*I am worried about dying*” and the highest correlation was seen for the item “*My eating habits have changed*”. For Assessment 2 “*I am worried about my appearance*” (CITC = 0.40) was the lowest and *“I have difficulty leaving the house”* (CITC = 0.80) was the highest for Part A. The corrected item-total correlation for Part B ranged from 0.25 to 0.64 for assessment 1 and 0.24 to 0.65 for assessment 2, reflecting mostly moderate internal consistency. The lowest score was observed for item “*I have hair loss*” (CITC = 0.25) for assessment 1 and “*I have night sweats*” for assessment 2 (CITC = 0.24). This indicated that the HM-PRO is well-balanced in both Part A and Part B, as no item carried too much weight.

### Test-Retest Reliability

The reproducibility of the HM-PRO score in repeated administration was tested. Patients completed the HM-PRO on two different occasions, at baseline (assessment 1) and a follow-up assessment after 7 days (assessment 2). The level of agreement was very strong for overall Part A: ICC = 0.93 (95% CI = 0.90–0.95), and Part B: ICC = 0.91 (0.88–0.93) (**Table 2**). The ICC for four domains of Part A ranged from 0.85 to 0.91, with social well-being scoring the lowest (ICC = 0.85, 95% CI = 0.79–0.89) and physical behavior showing the highest agreement (ICC = 0.91, 95% CI = 0.88–0.94). The correlation between the mean score of test and re-test was calculated by Spearman’s correlation coefficient (r_s_) (**Table 4**). The r_s_ ranged from 0.74 to 0.87, with Part A (r_s_ = 0.87) and Part B (r_s_ = 0.84) showing strong correlation. With respect to individual domains, physical behavior showed the highest correlation (r_s_ = 0.83), followed by emotional behavior (r_s_ = 0.82), eating and drinking (r_s_ = 0.75), but the lowest correlation for social well-being (r_s_ = 0.74).

**Table 2:** Test-retest reliability of the HM-PRO (n =150).

Domain/Scale (no. of items)	Assessment 1Mean (SD)	Range	Assessment 2Mean (SD)	Range	Mean Diff	r_s_	ICC (95% CI)	Alpha (α)
PB (7)	4.8 (3.6)	2–8	4.5 (3.7)	1–8	0.31	0.83	0.91 (0.88–0.94)	0.91
SW (3)	1.5 (1.6)	0–2	1.4 (1.6)	0–2	0.11	0.74	0.85 (0.79–0.89)	0.85
EB (11)	7.9 (4.5)	4–10	7.5 (4.8)	3.8–10.2	0.34	0.82	0.907 (0.87–0.93)	0.91
ED (2)	1.4 (1.4)	0–2	1.3 (1.3)	0–2	0.12	0.75	0.862 (0.81–0.90)	0.86
Part A (23)	15.4 (9.1)	8–22	14.6 (9.6)	6–21	0.81	0.87	0.926 (0.90–0.95)	0.93
Part B (17)	7.7 (5.2)	4–10	7.3 (5.1)	3–10	0.35	0.84	0.91 (0.88–0.93)	0.91

PB, physical behavior; SW, social well-being; EB, emotional behavior; ED, eating and drinking; r_s_, Spearman’s correlation coefficient; ICC, intra-class correlation coefficient.

With respect to agreement between individual items, ICC for all Part A items were greater than 0.7 and ranged from 0.73 to 0.94 (**Table 3**), with *“My sleeping pattern has changed”* scoring the lowest (ICC = 0.73, 95% CI = 0.62–0.80) and *“I have difficulty with work (or studies)”* showing the highest agreement (ICC = 0.94, 95% CI = 0.91–0.96). Further, the ICC for all the items in Part B ranged from 0.62–0.83. A total of four items had ICC lower than 0.7: *“I have/had fever”* (ICC = 0.62, 95% CI = 0.48–0.73); “*I have headaches*” (ICC = 0.66, 95% CI = 0.53–0.75); “I have stomach ache” (ICC = 0.69, 95% CI = 0.57–0.77); and “*I have infections (e.g., chest, lung, urinary, etc.)*” (ICC = 0.69, 95% CI = 0.57–0.77). The item with the highest agreement in Part B was “*I feel tired*” (ICC = 0.83, 95% CI = 0.77–0.88).

**Table 3:** Intra-class Correlation Coefficient (ICC) for individual items of Part A and Part B of the HM-PRO (n = 150).

Part A Items	ICC	95% CI		Sig	Part B Items	ICC	95% CI		Sig
		Lower	Upper				Lower	Upper	
PB1Walking	0.91	0.88	0.94	0.001	SS1Fever	0.62	0.48	0.73	0.001
PB2Selfcare	0.84	0.77	0.88	0.001	SS2Stomachache	0.69	0.57	0.77	0.001
PB3Phyactsports	0.83	0.76	0.88	0.001	SS3Energylevel	0.80	0.73	0.86	0.001
PB4Travelling	0.85	0.79	0.90	0.001	SS4Hairloss	0.75	0.65	0.81	0.001
PB5Leavingthehouse	0.81	0.73	0.86	0.001	SS5Tired	0.83	0.77	0.88	0.001
PB6Workorstudies	0.94	0.91	0.96	0.001	SS6Backpain	0.78	0.69	0.84	0.001
PB7Holidays	0.87	0.82	0.91	0.001	SS7Senseoftaste	0.80	0.72	0.85	0.001
SW1Socializing	0.81	0.73	0.86	0.001	SS8Breathing	0.80	0.73	0.86	0.001
SW2Personalrelationships	0.76	0.66	0.83	0.001	SS10Headaches	0.66	0.53	0.75	0.001
SW3Sexlife	0.91	0.85	0.94	0.001	SS11Constipation	0.79	0.71	0.85	0.001
EB1Burdentoothers	0.78	0.69	0.84	0.001	SS12Lumps	0.71	0.60	0.79	0.001
EB2Peoplejudgingme	0.77	0.68	0.84	0.001	SS13Bodypain	0.80	0.73	0.86	0.001
EB3Appearance	0.89	0.86	0.93	0.001	SS14Infections	0.69	0.57	0.77	0.001
EB4Distressed	0.79	0.71	0.85	0.001	SS15Nightsweats	0.81	0.74	0.87	0.001
EB5Anxious	0.76	0.67	0.83	0.001	SS16Diarrhoea	0.76	0.66	0.82	0.001
EB6Dying	0.85	0.79	0.90	0.001	SS17Nausea	0.83	0.76	0.88	0.001
EB7Confidence	0.77	0.68	0.84	0.001	SS18Chestpain	0.71	0.59	0.79	0.001
EB8Futurehealth	0.86	0.81	0.90	0.001					
EB9Sleepingpattern	0.73	0.62	0.80	0.001					
EB10Concentrating	0.79	0.72	0.85	0.001					
EB11Treatment	0.75	0.66	0.82	0.001					
ED1Appetite	0.87	0.82	0.91	0.001					
ED2Eatinghabits	0.80	0.72	0.85	0.001					

PB, physical behavior; SW, social well-being; EB, emotional behavior; ED, eating and drinking; SS, signs and symptoms.

Furthermore, since HM-PRO is developed to be used across different hematological malignancies and stages of disease, it is therefore important to test the reliability of the instrument across these patient groups. The patient data were grouped as per the disease diagnosis and stage of disease, and ICC was calculated for the two assessments 1 and 2 (t1 and t2) to test the agreement. With respect to the stage of disease, the ICC was greater than 0.7 for all the four domains and overall score of Part A and Part B (**Table 4**) confirming the reliability of the HM-PRO across the three stages of disease. The highest agreement was observed for physical behavior domain for “stable” (ICC = 0.92, 0.86–0.95) and “remission” (ICC = 0.91, 0.83–0.95) stage of disease, whereas emotional behavior (ICC = 0.93, 0.87–0.96) had the highest ICC for “progressing” stage of disease. For the overall scores of Parts A and B, ICC was greater than 0.8 showing strong agreement between the two assessments across three stages of disease (**Table 4**).

**Table 4:** Intra-class correlation coefficients of HM-PRO for the three-states of disease.

Domain/Scale	Stable		Remission		Progressing	
	ICC	95% CI	ICC	95% CI	ICC	95% CI
Physical Behavior	0.92	(0.86–0.95)	0.91	(0.83–0.95)	0.89	(0.80–0.94)
Social Well-being	0.75	(0.83–0.94)	0.90	(0.83–0.94)	0.88	(0.78–0.94)
Emotional Behavior	0.89	(0.82–0.94)	0.90	(0.83–0.94)	0.93	(0.87–0.96)
Eating and Drinking	0.91	(0.84–0.94)	0.74	(0.54–0.85)	0.88	(0.77–0.93)
Part A	0.92	(0.86–0.95)	0.92	(0.87–0.96)	0.93	(0.88–0.97)
Part B	0.89	(0.82–0.94)	0.91	(0.84–0.95)	0.92	(0.85–0.96)

ICC, intra-class correlation coefficient.

With respect to the reliability testing across disease diagnosis, the ICC was greater than 0.80 for the overall score of Parts A and B, for all the 10 diagnoses, confirming strong reliability. Further, ICC was greater than 0.70 for all for domains across 10 diagnoses, but 0.53 for “eating and drinking” domain, demonstrating that the HM-PRO possesses acceptable reliability in each one of the 10 hematological malignancy (**Table 5**).

**Table 5:** Intra-class correlation coefficient of HM-PRO across different hematological malignancies.

Disease Diagnosis	Part A Domains				Scales	
	Physical Behavior	Social Well-being	Emotional Behavior	Eating and Drinking	Part A	Part B
ALL	0.96	0.80	0.89	0.53	0.94	0.87
MPN	0.88	0.85	0.92	0.87	0.95	0.91
AML	0.77	0.85	0.85	0.81	0.81	0.84
ANHL	0.94	0.98	0.97	0.45	0.97	0.92
CLL	0.99	0.87	0.95	0.82	0.97	0.94
CML	0.74	0.83	0.83	0.93	0.82	0.91
HL	0.89	0.79	0.87	0.81	0.86	0.94
INHL	0.93	0.89	0.92	0.95	0.92	0.93
MDS	0.94	0.86	0.83	0.95	0.91	0.70
MM	0.94	0.71	0.86	0.93	0.93	0.95

ALL, acute lymphoid leukemia; AML, acute myeloid leukemia; CLL, chronic lymphoid leukaemia; CML, chronic myeloid leukemia; MM, multiple myeloma; INHL, indolent non-Hodgkin lymphoma; ANHL, aggressive non-Hodgkin lymphoma; HL, Hodgkin lymphoma; MDS, myelodysplastic syndrome; MPN, myeloproliferative neoplasm.

### Individual Item Correlation Between the Two Assessments

Following confirmation of the internal consistency and test-retest reliability, Spearman’s rank correlation coefficients were calculated for individual items in Part A and Part B, between the assessments 1 and 2 (t1 and t2). With respect to Part A, the correlation coefficient (r_s_) ranged from 0.58 to 0.86, showing a moderate to a strong relationship. The lowest r_s_ value was observed for the item “*My eating habits have changed”* (r_s_ = 0.58). During treatment, patients experienced frequent changes due to chemotherapy, especially in the *“sense of taste”*. Because of this the lower correlation can be justified for *“eating and drinking”* habits in the recall period “today”. With respect to individual items in Part B, the correlation coefficient ranged from 0.49 to 0.72. The lower range is expected because of two reasons: first, the signs and symptoms change very frequently; second, the recall period of Part B is the last 3 days, and patients completed the second assessment after 7 days. In general, all the items in Part A and Part B showed a moderate to a strong correlation, demonstrating further confirmation of the reliability of HM-PRO.

## Discussion

There are several health-related QoL instruments which are currently used in hematology (Goswami et al., 2019a). In choosing an instrument to meet the underlaying goals of assessing patient-reported outcomes, it is imperative to ascertain that such instrument is valid and reliable. The HM-PRO has been developed as a composite measure combining HRQoL and Symptoms scale for use in both clinical practice and research (Goswami et al., 2016). Therefore, possessing a good reliability not only signifies the internal validity but also ensures that the scores obtained in a setting is representative and stable over time, without which the results and the conclusions may mislead treatment decisions, particularly in a daily clinical practice setting. Evaluating the performance of an intervention may be under or overestimated with an unreliable instrument. This study focused on evaluating the reliability of the newly developed HM-PRO for its two Parts (A and B), as well as the four individual domains of Part A.

One of the main objectives of using PROs in clinical practice setting is to promote patient-centered care [210]. PROs can be used on a “group-level” or on “individual-level”. On the group-level, PROs can be used for treatment decision-making and screening of patients conditions, and on the individual-level they can be used to improve clinician-patient communication or detecting problems and improve patient outcomes or patient management [251, 252, 437–440]. The results suggest that both the scales and the individual domains of HM-PRO are homogenous and a strong level of agreement between test and retest scores. Hence, HM-PRO can be confidently used at both the group as well as individual level.

The concept of HRQoL is very subjective. Every patient has different perspective toward their own HRQoL. What is important to patients in terms of HRQoL issues may differ from individual to individual. Although medicines taken during the treatment might have the same mechanism of action, they may affect each patient differently. Some have high tolerance to pain whereas others may find it extremely difficult to cope with the slightest pain. With a strong evidence of reliability of HM-PRO, it can be used as a patient management tool for monitoring a patient’s condition over time on an individual basis.

The HM-PRO has been developed using both classical test theory as well as modern and sophisticated technique, i.e., item response theory, in particular Rasch modelling. A recent article published on Rasch measurement theory approach reflects how a PRO can be used to identify aspects of HRQoL which are important to patients and the aspects which they can benefit from the new treatment (Browne and Cano, 2019). However, a PRO to be used in important decision making has to be reliable and must have followed robust development methodology. The HM-PRO has the potential to be used as an aid in treatment decisions during routine clinical practice following further research to demonstrate such a clinical utility.

One of the main barriers to implementation of the PROs in routine clinical practice is the selection of a HRQoL instrument and interpretation of its scores. As identified by a systematic review, there are several HRQoL instruments which are currently used in hematology (Osborne et al., 2012). Some are disease specific instruments which have been recently developed for example MyPOS (Osborne et al., 2015) for multiple myeloma patients and AML-QoL (Buckley et al., 2020) for patients with acute myeloid leukemia, and there are other instruments which have been developed for general oncology with additional disease specific modules. Such a plethora of instruments discourages their use in the clinical practice mainly due to difficulty in selection and lack of understanding on how to calculate the scores and their interpretation. Hence, the HM-PRO as a generic tool being applicable to all hematological malignancies with good discriminant validity can help to overcome one of the major barriers for the use of PROs in routine clinical practice. It can be used across different hematological malignancies, while being sensitive to individual diagnosis as well. Furthermore, availability of a single user-friendly PRO would be far more welcoming by the clinician to use in their daily clinical practice to aid treatment decision making than a dozen from which to choose for each patient presenting a different hematological malignancy.

## Conclusion

The findings from this study clearly indicate that the HM-PRO possesses very good reliability rendering it an instrument with potential of being able to play an important clinical role; but that future research is needed to demonstrate the clinical utility of using the HM-PRO in routine practice to help clinicians make treatment decisions. The extensive reliability testing described in this study supports the generic nature of the HM-PRO for use in hematological malignancies in both routine clinical practice, as well as in research and its ability to be used as a management tool for monitoring a patient’s condition over a period of time.

## Data Availability Statement

The raw data supporting the conclusions of this article will be made available by the authors, without undue reservation.

## Ethics Statement

The studies involving human participants were reviewed and approved by NRES South West Bristol, UK (ref 14/SW/0033). The patients/participants provided their written informed consent to participate in this study.

## Author Contributions

PG collected the data, developed the analysis policy, liaised with hospitals for patient recruitment, analyzed the data, interpreted results, and wrote the first draft of the manuscript. RE contributed to data collection as a patient research partner and reviewed the draft manuscript. SS generated the original idea, developed the study protocol, supervised the study, liaised with study centers as part of patient recruitment, developed the analysis policy, interpreted results, and reviewed the draft manuscript. EO and TI contributed to the design of the study, interpreted results, and reviewed the draft manuscript. JK, AF, DJ, MK, SA-I, MO, MA-O, GC, SM, CL, and MA-O contributed to patient recruitment from their respective center and reviewed the draft manuscript. All authors contributed to the article and approved the submitted version.

## Funding

The study was funded by the European Hematology Association Scientific Working Group “Quality of life and Symptoms” through unrestricted grants from Novartis, Bristol Myers Squib and Sanofi. The funders had no role in study design, data collection and analysis, decision to publish, or preparation of the manuscript.

## Conflict of Interest

The authors declare that the research was conducted in the absence of any commercial or financial relationships that could be construed as a potential conflict of interest.
